# Impact of *Helicobacter pylori* infection on fluid duodenal microbial community structure and microbial metabolic pathways

**DOI:** 10.1186/s12866-022-02437-w

**Published:** 2022-01-15

**Authors:** Tadashi Maeda, Hiroaki Zai, Yuto Fukui, Yoshifumi Kato, Eri Kumade, Toshiyasu Watanabe, Norihiro Furusyo, Hitoshi Nakajima, Kazuho Arai, Yoshikazu Ishii, Kazuhiro Tateda, Yoshihisa Urita

**Affiliations:** 1 Department of General Medicine and Emergency Care, Toho University School of Medicine, 6- 11-1 Omori-nishi, Ota-ku, 143-8541 Tokyo, Japan; 2 Department of Microbiology and Infectious Diseases, Toho University School of Medicine, 5- 21-16 Omori-nishi, Ota-ku, 143-8540 Tokyo, Japan; 3 Department of General Medicine, Kyushu University, Fukuoka, Japan

**Keywords:** *Helicobacter pylori*, Duodenal microbiota, LEfSe, KEGG, Microbial metabolic pathway

## Abstract

**Background:**

The bioactivities of commensal duodenal microbiota greatly influence the biofunction of hosts. We investigated the role of *Helicobacter pylori* infection in extra-gastroduodenal diseases by determining the impact of *H. pylori* infection on the duodenal microbiota. We sequenced 16 S rRNA genes in samples aspirated from the descending duodenum of 47 (male, 20; female, 27) individuals who were screened for gastric cancer. Samples were analysed using 16 S rRNA gene amplicon sequencing, and the LEFSe and Kyoto Encyclopaedia of Genes and Genomes methods were used to determine whether the duodenal microflora and microbial biofunctions were affected using *H. pylori* infection.

**Results:**

Thirteen and 34 participants tested positive and negative for *H. pylori*, respectively. We identified 1,404 bacterial operational taxonomic units from 23 phyla and 253 genera. *H. pylori infection changed the relative mean abundance of three phyla (Proteobacteria, Actinobacteria, and TM7) and ten genera (Neisseria, Rothia, TM7-3, Leptotrichia, Lachnospiraceae, Megasphaera, F16, Moryella, Filifactor, and Paludibacter).* Microbiota features were significantly influenced in *H. pylori*-positive participants by 12 taxa mostly classified as *Gammaproteobacteria*. Microbial functional annotation revealed that *H. pylori* significantly affected 12 microbial metabolic pathways.

**Conclusions:**

*H. pylori* disrupted normal bacterial communities in the duodenum and changed the biofunctions of commensal microbiota primarily by upregulating specific metabolic pathways. Such upregulation may be involved in the onset of diseases associated with *H. pylori* infection.

**Supplementary Information:**

The online version contains supplementary material available at 10.1186/s12866-022-02437-w.

## Background

*Helicobacter pylori* infection can cause chronic gastritis or peptic ulcers and is associated with the development of certain gastric cancers [1]. Recent epidemiological findings suggest that the prevalence of cardiovascular disease, haematological disease, neurodegenerative disease, liver disease, and metabolic syndrome is high in patients with *H. pylori* infection [2–6]. However, causal relationships between the pathogenesis factors in *H. pylori* infections and various other extra-gastroduodenal diseases remain obscure [4, 7, 8].

The theory that the bioactivities of commensal gut microbiota greatly influence host biofunction has attracted considerable attention in elucidating the pathophysiology of various diseases. In addition, the duodenum plays a key role in the crosstalk between the gut and the central nervous system, particularly as the release of brain-gut hormones and neurotransmitters in the small intestine, including the duodenum, are regulated by eating stimuli and information arising from the intraluminal environment. These hormones regulate widespread biofunctions, such as metabolism, biosynthesis, feeding behaviour, and gastrointestinal functions [9, 10].

Schulz et al. [11] found that *H. pylori* infection alters the duodenal microbiota based on evidence from reverse-transcribed 16 S rRNA and that the same results were derived from duodenal biopsies and aspirates. These findings suggest that alteration in the duodenal microbiota induced by *H. pylori* infection is related to the onset of various extra-gastroduodenal diseases. This is because some degradation products of digestion, attributed to duodenal microbial biofunction, act as chemical effectors on host biofunctions [12, 13].

Therefore, we aimed to elucidate the impact of *H. pylori* infection on the structure of commensal duodenal microbiota and their biofunctions using conventional analyses of microbial taxonomic diversity and the novel linear discriminant analysis (LDA) effect size (LEfSe) algorithm method to discover metagenomic biomarkers that could explain differences among microbial communities [14]. We also applied metagenomic functional prediction using the Kyoto Encyclopaedia of Genes and Genomes (KEGG) database to infer the microbial genetic features associated with biological functions and metabolic pathways [15–17], which facilitate the extraction of specific genetic information related to microbial biofunction from a chaotic metagenomic bin.

## Results

Thirteen and 34 samples were *H. pylori*-positive and negative, respectively. Considering the Kimura–Takemoto classification of endoscopic atrophy, 30 of the 34 participants (88.2%) in the *H. pylori*-negative group had C0 (non-atrophy)–C2 atrophy, and most of the remaining four participants had a history of *H. pylori* eradication therapy. In contrast, C0–C2 atrophy was observed in five of the 13 participants (38.5%) in the *H. pylori*-positive group (Supplementary Information 1). These results indicate that gastric acid secretion decreased in the *H. pylori*-positive group.

MiSeq sequencing produced 3,312,084 reads with a mean of 70,470 ± 14,833 sequences per sample. These analyses were based on a rarefied table determined from 8,804 gene reads per sample. We identified 1,404 bacterial operational taxonomy units (OTU) from 23 phyla and 253 genera. The numbers of bacterial OTU per participant were 263.86 ± 78.46 and 284.53 ± 67.20 in the *H. pylori*-negative and -positive groups, respectively. These sequence data are available in the DDBJ Sequence Read Archive under the accession number DRA011815 (DRX275800 to DRX275846).

### Association of *H. pylori* with microbial diversity

Neither α- nor β-diversity significantly differed between the *H. pylori*-positive and -negative groups (Supplementary Fig. 2).

### Influence of *H. pylori* on bacterial community structures

**Fig. 1:**
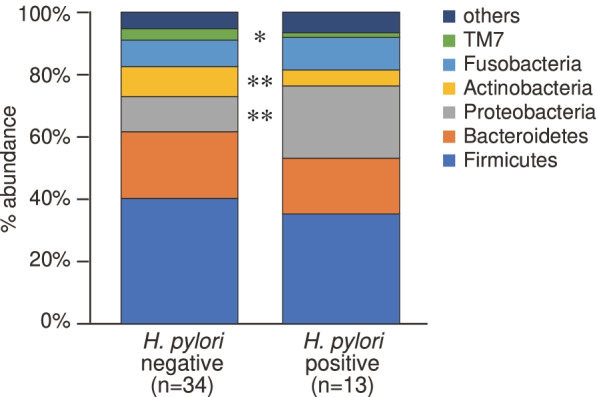
Differences in bacterial community structures at phylum level in *Helicobacter pylori*-positive and -negative groups.&nbsp;*P < 0.05 and **P < 0.01 (Welch’s *t*-tests)

Figure 1 shows bacterial community structures at the phylum level. The relative mean abundance of *Actinobacteria* (negative 9.5%: positive 5.1%) and *TM7* (negative 3.6%: positive 1.5%) was significantly higher in the *H. pylori*-negative group than in the *H. pylori*-positive group. In contrast, the abundance of *Proteobacteria* (negative 11.3%: positive 23%) was significantly higher in the *H. pylori*-positive group, and *Acidobacteria* and *Planctomycetes* were evident only in the *H. pylori*-negative group. The relative mean abundance of 14 phyla did not significantly differ between the *H. pylori*-negative and -positive groups. The results for the other four phyla were invalid for statistical analysis (Supplementary Information 2).

The relative mean abundance of ten genera significantly differed between the *H. pylori*-negative and -positive groups (Table 1). Only the relative abundance of *Neisseria* was significantly higher in the *H. pylori*-positive group. In addition, the Mann–Whitney *U* test was used to compare the *H. pylori*-positive and *H. pylori*-negative groups of *Neisseria*. The median was 0.025 in the negative group, 0.140 in the positive group, and the P-value was 0.002. The relative abundance of the other nine genera (*Rothia*, [unknown order] TM7-3, *Leptotrichia*, [unknown genus] Lachnospiraceae, *Megasphaera*, [unknown genus] F16, *Moryella*, *Filifactor*, and *Paludibacter*) was significantly higher in the *H. pylori*-negative group. Furthermore, 188 and 143 genera were detected in the *H. pylori*-negative and -positive groups, respectively. These differences were attributed to the different microbial community structures of each group. Specifically, 60 and 15 genera were found only in the *H. pylori*-negative and -positive groups, respectively. *Helicobacter* was detected only in the *H. pylori*-positive group, with an abundance of 2.79 ± 6.82% (Supplementary Information 3).

**Table 1:** Difference in the relative mean abundance of taxa at genus level in *Helicobacter pylori*-positive and -negative groups

Genus	Relative mean abundance (%) ± SD		
	*H. pylori*	*H. pylori*	P^a^
	negative (n = 34)	positive (n = 13)	
*Neisseria*	4.76 ± 5.93	11.74 ± 7.10	<0.01
*Rothia*	6.83 ± 7.44	1.81 ± 1.36	<0.001
{Unknown Order} TM7-3	2.74 ± 3.54	0.80 ± 0.89	<0.01
*Leptotrichia*	2.06 ± 1.84	1.18 ± 1.02	<0.05
{Unknown Genus} Lachnospiraceae	0.90 ± 0.84	0.33 ± 0.30	<0.01
*Megasphaera*	0.72 ± 0.76	0.38 ± 0.33	<0.05
{Unknown Genus} F16	0.49 ± 0.60	0.21 ± 0.23	<0.05
*Moryella*	0.32 ± 0.39	0.11 ± 0.13	<0.05
*Filifactor*	0.28 ± 0.45	0.09 ± 0.16	<0.05
*Paludibacter*	0.04 ± 0.06	0.01 ± 0.02	<0.05

Ten taxa with significant differences among 253 detected are shown. Some taxa that could not be identified at genus level were classified at higher levels. Notations in parentheses indicate classification level. ^a^Calculated using Welch’s *t*-test

### Influence of *H. pylori* on biologically relevant features

**Fig. 2:**
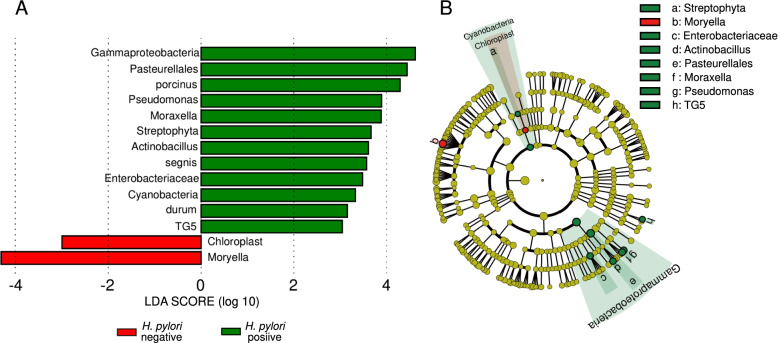
LEfSe analysis of differences in biologically relevant features between *Helicobacter pylori*-positive and -negative groups.&nbsp;Green and red: *H. pylori*-positive and -negative, respectively. (**A**) Rank of effect size of each taxa. (**B**) Taxonomic cladogram considering hierarchy and systematic closeness&nbsp;porcinus: *Streptococcus porcinus*, segnis: *Haemophilus segnis*, durum: *Paenibacillus* *durum*

The LDA score derived from LEfSe analyses indicated that 12 taxa significantly influenced the biological features of the duodenal microbiota in the *H. pylori*-positive group (Fig. 2 A). These 12 taxa comprised three phyla (*Streptophyta*, *Cyanobacteria*, and *TG5*), one class (*Gammaproteobacteria*), one order (*Pasteurellales*), one family (*Enterobacteriaceae*), three genera (*Pseudomonas*, *Moraxella*, and *Actinobacillus*), and three species (*Streptococcus porcinus*, *Haemophilus segnis*, and *Paenibacillus durum*) of bacteria. Figure 2B shows the information on biological classification. Six of these taxa belonged to the class *Gammaproteobacteria* (phylum *Proteobacteria*): *Pasteurellales*, *Pseudomonas*, *Moraxella*, *Actinobacillus*, *H. segnis*, and *Enterobacteriaceae*. *Moraxella* and *H. segnis* are notable intraoral bacteria. Two taxa, *S. porcinus* and *P. durum*, belonged to the class *Bacilli* (belonging to *Firmicutes*). These results suggest *Gammaproteobacteria* can affect duodenal microbial features.

### Influence of *H. pylori* on duodenal microbial biofunctions

Among 327 investigated KEGG pathways (Supplementary Information 4), 163 were metabolic. The ko-abundance of 12 of these metabolic pathways significantly differed in the presence or absence of *H. pylori* infection (Table 2), and the ko-abundance of nine of these 12 was significantly greater in the *H. pylori*-positive group (synthesis and degradation of ketone bodies, tryptophan metabolism, ether lipid metabolism, linoleic acid metabolism, alpha-linolenic acid metabolism, biotin metabolism, carotenoid biosynthesis, phenylpropanoid biosynthesis, biosynthesis of terpenoids and steroids). This suggests that *H. pylori* generally upregulates metabolic functional activities in duodenal microbes. The remaining pathways comprised those associated with genetic information processing (n = 16), environmental information processing (n = 21), cellular processes (n = 19), organismal systems (n = 48), and human diseases (n = 60). Among these 164 pathways, 18 significantly differed between the individuals with and without *H. pylori*. However, it was impossible to infer whether these pathways were functioning for microbial biofunctions and affecting biofunctions of the host.

**Table 2:** Differences in KEGG metabolic pathways between *Helicobacter pylori*-positive and -negative groups

Class	KEGG pathway		Median ko-abundance		
	(ko number)	Pathway	*H. pylori*	*H. pylori*	
			negative (n = 34)	positive (n = 13)	P^b^
M	ko00072^†^	Synthesis and degradation of ketone bodies	1335885.8	1823878.4	<0.05
M	ko00380^†^	Tryptophan metabolism	2254680.9	2759405.6	<0.05
M	ko00510	N-glycan biosynthesis	907554.4	700748.0	<0.05
M	ko00565^a^	Ether lipid metabolism	150966.1	394815.2	<0.001
M	ko00571	Lipoarabinomannan (LAM) biosynthesis	397200.0	191311.5	<0.05
M	ko00591^a^	Linoleic acid metabolism	85148.3	389478.0	<0.001
M	ko00592^a^	alpha-Linolenic acid metabolism	132544.9	405656.4	<0.001
M	ko00780^a^	Biotin metabolism	15103400	19564468	<0.05
M	ko00906^a^	Carotenoid biosynthesis	129310.3	510048.5	<0.01
M	ko00940^†^	Phenylpropanoid biosynthesis	348411.2	598460.1	<0.01
M	ko01053	Biosynthesis of siderophore group nonribosomal peptides	2025414.2	1127312.8	<0.01
M	ko01062^a^	Biosynthesis of terpenoids and steroids	92469.4	428251.7	<0.01

Twelve metabolic pathways that significantly differed among 327 detected pathways. M, metabolism. ^a^Most abundant pathways in *H. pylori-*positive group. ^b^Calculated using Mann–Whitney *U* tests

## Discussion

Although a causal relationship has long been suspected between *H. pylori* infection and cardiovascular disease, haematologic disease, and metabolic syndrome, the roles of commensal microbiota in these diseases have remained obscure [3]. This study found that *H. pylori* infection significantly influenced the relative abundance of three phyla and ten genera in the duodenal microbiota and that the altered duodenal microbiota was characterised by increased *Neisseria* abundance and an enhanced impact of *Gammaproteobacteria.* The abundance of multiple commensal microbial metabolic pathways was also significantly altered, suggesting that *H. pylori* altered aspects of microbial metabolites that may affect host biofunctions. Many studies have investigated the gut microbiota before and after therapy for various diseases [6, 18]. Although many comparative studies on the gut microbiota have associated differences in the gut microbiota with certain diseases, the results remain inadequate, especially for factors originating in different microbiota that are substantial etiological effectors [5].

Although a study comparing biopsied gastric tissue with and without *H. pylori* reported differences in diversity [11], the present α- and β-diversity analyses in this limited sample size revealed no significant differences between *H. pylori*-positive and -negative groups (Supplementary Fig. 2). Although the results of the diversity analyses indicate no differences, this does not necessarily indicate that an identical abundance or representation of bacterial species exists in each group. In fact, the duodenal bacterial community structures differed at the phylum level between the two groups, with a greater abundance of *Proteobacteria* and a lower abundance of *Actinobacteria* and *TM7* (*Saccharibacteria*) in the *H. pylori*-positive group. However, since the phylum to which *H. pylori* belongs has recently been changed from *Proteobacteria* to the newly established *Epsilonbacteraeota* [19], the results for the relative abundance of the bacterial community structure at the phylum level may change. The bacterial community structure at the genus level and LEfSe results suggested that *H. pylori* infection altered the microbial features by increasing the abundance of *Neisseria* and enhancing the impact of *Gammaproteobacteria* in the duodenum (Table 1; Fig. 2). These results appear to be inconsistent, as the abundance of *Neisseria* was significantly higher in the positive group in the genus-level community structure analysis, but not significantly different in the phylum and genus-level LEfSe analysis. This inconsistency likely originates from the different methods used and the purpose of each analysis. The results for the bacterial community structure were derived by relating the relative abundance of the number of reads for each taxon (or OTUs) to the number of reads for the entire sequence. These results do not indicate the actual number of bacteria because the method aligns the DNA concentrations between samples to equal concentrations. LEfSe is designed to increase the detection power compared to community structure analysis, which compares simple ratios [14]. Therefore, the two methods yield different results.

The increase in *Neisseria* in the duodenum is probably related to the gastric acid output owing to atrophic gastritis induced by *H. pylori*. Intraoral indigenous bacteria in the genus *Neisseria* are not generally highly pathogenic, except for *Neisseria gonorrhoeae* and *Neisseria meningitidis*, which cause gonorrhoea and meningitis, respectively [20]. However, excessive *Neisseria* proliferation in the duodenum may be pathogenic through changes in microbial community structure [21, 22]. The subclass *Gammaproteobacteria* comprises several medically important bacteria, such as 

### *Enterobacteriaceae*, *Vibrionaceae*, and *Pseudomonadaceae*

Many studies on the relationships between *H. pylori* infections and extra-gastric diseases have identified increased short-chain fatty acid (SCFA) production induced by the proliferation of *Bacteroidetes* [23]. These SCFAs induce the release of gut hormones such as peptide YY and glucose like peptide-1, activation of host metabolic pathways, mucosal immune response, and inflammation [18, 23, 24]. This study revealed that *H. pylori* did not significantly change the abundance of *Bacteroidetes* in the duodenum (Fig. 1). The LDA scores also indicated that taxa belonging to *Bacteroidetes* did not significantly affect duodenal microbial features (Fig. 2). These findings were consistent with previous analyses of duodenal aspirates [10]. Although the hypothesis that increased SCFA production causes various diseases is attractive, SCFAs are generated primarily through the fermentation of nonhost-digestible dietary fibres by the colonic microbiota. Therefore, other factors associated with upper gastrointestinal microbial functions should be considered.

The KEGG pathway analysis showed that 12 bacterial metabolic pathways were affected by the presence or absence of *H. pylori* infection. Two pathways that were upregulated in the *H. pylori*-positive group, synthesis and degradation of ketone bodies (ko00072) and ether lipid metabolism (ko00565), are important for degradation of fatty acids, butyrate and acetic acid synthesis, and the production of phosphocholine or seminolipid, which functions in the maintenance of mucosal integrity and immune homeostasis [25, 26].

Notably, the ko-abundance of the tryptophan metabolic pathway (ko00380) was significantly greater in the *H. pylori*-positive group, suggesting that an abnormal tryptophan supply from the intestine impaired serotonin production. Serotonin is a paracrine messenger expressed primarily in enterochromaffin cells and enteric neurons. This information would help to clarify the causal relationship between *H. pylori* infection, the duodenal microbiota, and the pathophysiology of functional dyspepsia [27–29]. In addition, serotonin production issues may alter local serotonin concentrations in portal blood, which can also affect the gut-liver axis [30].

The pathways of linoleic (ko00591) and α-linolenic (ko00592) acid metabolism were also upregulated in the *H. pylori*-positive group (Table 2). Such upregulation may cause an imbalance between ω-3 and ω-6 fatty acids and affect the arachidonic acid cascade associated with inflammation [31]. The biotin metabolic pathway (ko00780) was also upregulated in the *H. pylori*-positive group. Bacteria synthesise biotin, which is an indispensable essential cofactor for fatty acid biosynthesis. Vitamin A production may also be affected by *H. pylori* because the biosynthetic pathway of the vitamin A precursor, carotenoid (ko00906), was upregulated in the *H. pylori*-positive group [32, 33]. The phenylpropanoid biosynthesis pathway (ko00940) was also upregulated significantly in this group. However, the physiological significance of this upregulation in humans is difficult to determine because the roles of metabolites (such as chavicol, eugenol, lignin) originating in this pathway have not been fully elucidated. The terpenoid and steroid biosynthesis pathway (ko01062) was upregulated in the *H. pylori*-positive group. This could extensively affect host functions because terpenoids are steroid precursors and closely related to cytochrome P450 that functions as an oxidase in terpenoid biosynthesis [34, 35].

Yap et al. [36] found 45 upregulated and 551 downregulated serum metabolites 18 months after *H. pylori* eradication. The affected metabolites were mapped to various biochemical pathways, including tryptophan metabolism, biosynthesis of unsaturated fatty acids, and linoleic acid metabolism. Although whether these alterations affect host biofunctions remains obscure, our findings confirmed that the metabolomic findings reported by Yap et al. originated from microbial metabolic pathways affected by *H. pylori* infection. Notably, KEGG analysis is only a prediction due to the presence of DNA. Transcriptome analysis will be needed to determine if these metabolic pathways are indeed upregulated.

This study has several limitations and issues. First, we could not exclude the possibility that some subjects in the *H. pylori*-negative group might have already experienced significant changes in the structure and biofunctions of the commensal duodenal microbiota due to previous *H. pylori* infections. In fact, nine patients with a history of infection and eradication were included in the *H. pylori*-negative group (Supplementary Information 1). To eliminate this concern, it is necessary to analyse changes in microbial features before and after *H. pylori* eradication therapy in the same subject.

In addition, we could not comprehensively evaluate the effect of gastric acid on the duodenal microbiota because we did not quantify gastric acid secretion. The extent of gastric mucosal atrophy caused by *H. pylori* infection depends on various factors, such as age, duration of infection, differences between individual immune responses, and the number of bacteria. The gastric acid output depends on the extent of gastric mucosal atrophy, and the extent of atrophic gastritis is closely related to a history of *H. pylori* infection [37]. In fact, the endoscopic findings in this study indicated that an extended atrophic change was likely to be observed in the *H. pylori*-positive group. Moreover, the duodenal microbiota might be affected by a decrease in gastric acid output.

Another limitation is that contamination of the gastric microbiota could not be completely ruled out because of the sampling method and due to the lack of bacterial culture. A concern has been raised that aspirate samples include only floating microbiota, which may have little to do with host biofunctions, and that the microbiota originating from biopsy samples (mucosa-associated microbial community structure) inhabit the mucosa [38, 39]. Our findings suggest that microbial metabolite production may fluctuate depending on changes in commensal duodenal microbiota and this phenomenon may affect host biofunctions. These action mechanisms may not depend on areas inhabited by microbes, such as duodenal juice or mucosa, because microbial metabolites act as chemical effectors. This study focused on changes in duodenal bacterial flora caused by the presence of *H. pylori* rather than direct changes in the duodenal bacterial flora.

Finally, differential abundance (DA) analysis methods for microbiome data are controversial in terms of consistency and reliability. For example, some have pointed out that the false discovery rate could sometimes not be controlled in LEfSe analysis, which was used in this study [40]. The problem originates in the biases due to differences in sampling fractions among collected samples, and it would be difficult to correct the biases adequately. Recently, the Analysis of Compositions of Microbiomes with Bias Correction (ANCOM-BC) has been proposed as a solution to overcome this shortcoming and is considered having a potential as a more reliable DA analysis method for microbiome data [41].

In conclusion, *H. pylori* infection changed the aspects of the microbiota in the descending part of the duodenum. This dysbiosis, characterised primarily by the upregulation of microbial metabolic pathways, altered commensal microbial biofunctions, which may affect host biofunctions. The gut microbiota can be regarded as an independent organ within the gut lumen, and an investigation of biofunctions originating from this “commensal bacterial organ” would help elucidating the aetiology of various diseases.

## Methods

### Participants

This study included 20 males and 27 female patients living in Ishigaki Island, Okinawa, Japan (mean age: 58.8 ± 11.3 years), who were screened for gastric cancer. We obtained information from all participants about treatment with gastric acid inhibitors, antibiotics, and medical history of *H. pylori* eradication (Supplementary Information 1). Patients treated with antibiotics within 4 weeks before sampling were excluded. To establish a valid standard deviation that could provide a 95% confidence interval of the mean value, the sample size of *H. pylori*-negative participants was over 30. The obtained data were used as normal control data for statistical analysis. We collected duodenal aspiration in order of examinee, and the sample size of *H. pylori*-positive participants was settled when the sample size of participants in the negative group reached 30 and beyond. The study protocol (Supplementary Fig. 1) was implemented under the approval of the Ethics Committee of the Toho University School of Medicine (authorisation number: A16080), in accordance with current good clinical practice and the Declaration of Helsinki (2013). All participants provided written informed consent to participate before enrolment.

### Patient and public involvement

The design of this study proceeded without public involvement. Patient involvement was restricted to sample collection at the time of enrolment. Patients were neither consulted to interpret the results nor invited to contribute to the writing or editing of this article.

### Collection of duodenal fluids and esophagogastroduodenoscopy

Duodenal fluids were collected from the descending part of the duodenum using a PW-2 L-1 fluororesin tube (Olympus, Tokyo, Japan) under standard video endoscopy with the Olympus GIF-XQ260 or GIF-XP260N video gastroscope (Olympus, Tokyo, Japan). The tube was sterilised and changed for each patient. Duodenal fluid was aspirated immediately after injecting 5 mL of saline into the descending part of the duodenum, and the aspirate was immediately cryopreserved at −80 °C. One certified endoscopist (HZ) conducted all endoscopic procedures and sampling to avoid bias. The endoscopic findings were recorded simultaneously, and the extent of atrophic gastritis was evaluated in accordance with Kimura–Takemoto classification for endoscopic atrophy [42].

### Extraction of genomic DNA

Genomic DNA (gDNA) was extracted from duodenal fluid using PowerFecal DNA Isolation Kit (Mo Bio Laboratories, Inc., Carlsbad, CA, USA) as described in the protocol provided by the manufacturer.

### Identification of *H. pylori*

We applied conventional nested polymerase chain reactions (PCR) of extracted gDNA [43] and then sequenced amplicons to verify the presence of *H. pylori*. The participants were then assigned to groups based on the presence or absence of *H. pylori*.

### Sequencing

Sequencing libraries were prepared for the Illumina MiSeq platform (Illumina, San Diego, CA, USA). The V3 and V4 regions of the 16 S rRNA gene were targeted using the primer pair 341 F (5′-CCTACGGGNGGCWGCAG-3′) and 806R (5′-GACTACHVGGGTATCTAATCC-3′) [44], and a 16 S rRNA gene library was prepared for sequencing as described in the protocol provided by the manufacturer (https://support.illumina.com/documents/documentation/chemistry_documentation/16s/16s-metagenomic-library-prep-guide-15044223-b.pdf). The PCR amplicons were purified using Wizard SV Gel and PCR Clean-Up System (Promega, St. Louis, MO, USA), then sequenced and quantified using the MiSeq system [45].

### Bioinformatic and statistical analyses

Sequencing data were processed using CLC Genomic Workbench 10.0.1 and CLC Microbial Genomics Module 2.5 (Qiagen, Hilden, Germany). Overlapping paired-end reads were merged and trimmed, and chimeric reads were filtered using default parameters. The remaining reads were clustered into OTU with 97% identity using the Greengenes database (version 13.5) as the reference [46]. We evaluated bacterial diversity by calculating α- and β-diversity from rarefied OTU tables. The α-diversity was evaluated using richness based on the number of OTU and evenness appraised using the Shannon diversity index [47, 48]. Statistical analyses were performed using EZR, a graphical user interface for R [49]. β-diversity was evaluated based on the OTU Table [50] as the unweighted UniFrac distance can distinguish dissimilarities between microbial profiles of two samples. The β-diversity results were analysed via permutational multivariate analyses of variance (PERMANOVA) using the CLC Genomics Workbench 10.0.1. The relative abundances of phyla and genera in the *H. pylori*-positive and -negative groups were compared based on unrarefied OTU tables using Welch’s *t*-tests in Microsoft Excel for Windows 10.

The LEfSe algorithm can identify genomic taxa with a relative abundance that differs between groups. We computed LEfSe using the Galaxy web application and workflow framework (https://huttenhower.sph.harvard.edu/galaxy/) to support high-dimensional class comparisons with a focus on metagenomic analysis.

The biofunctions of the duodenal microbiota were inferred via metagenomic functional annotation. The OTU abundance table was uploaded to the Piphillin server (https://piphillin.secondgenome.com/) with the KEGG Orthology (KO) database as the reference genomic database [51]. Thereafter, KEGG pathways were identified based on the gene information in the OTU, enabling the interpretations of high-level biofunctions of the microbiota.

The KEGG pathway analysis results identified the ko-abundances that corresponded to the abundance of specific KEGG pathways and quantitatively represented microbial biofunction characteristics. Then, pathways classified under metabolism were investigated in detail because the gut microbiota could be regarded as an independent organ that produces various metabolites with metabolic functions in the gut lumen [13]. Each ko-abundance was compared between the *H. pylori*-positive and -negative groups using Mann–Whitney *U* tests in Microsoft Excel for Windows 10. All values with *P <* 0.05 were considered statistically significant.

## Supplementary Information

**Additional file 1.**

**Additional file 2.**

**Additional file 3.**

**Additional file 4.**

**Additional file 5.**

**Additional file 6.**

## Data Availability

Data are available in a public, open access repository. Sequence data are available from DDBJ (https://www.ddbj.nig.ac.jp/) under the accession number DRA011815 (DRX275800 to DRX275846).

## Abbreviations

DA: DA, Differential abundance
KEGG: Kyoto Encyclopaedia of Genes and Genomes
KO: KEGG Orthology
LDA: Linear discriminant analysis
OTU: Operational taxonomy units
PCR: Polymerase chain reactions
PERMANOVA: Permutational multivariate analyses of variance
SCFA: Short-chain fatty acid
