# Supplementary material for: Impact of Helicobacter pylori infection on fluid duodenal microbial community structure and microbial metabolic pathways
Source: BMC Microbiol. 2022 Jan 15;22:27. doi: 10.1186/s12866-022-02437-w (PMC8760755; doi:10.1186/s12866-022-02437-w)
Supplement: Supplementary file 1 — Additional file 1. [file 12866_2022_2437_MOESM1_ESM.docx]

**Supplementary Information 1: Overview of subject information**

Past Kimura–Takemoto

Subject *H. pylori* history of Endoscopic classification of Acid

No. (PCR test) *H. pylori* diagnosis endoscopic Age^1^ Gender^2^ suppressive

eradication in the stomach atrophy therapy^3^

1 - - Superficial gastritis C0 52 male none

2 - - Superficial gastritis C0 57 male none

3 - - Superficial gastritis C0 72 female lansoprazole 15mg (due to gastritis)

4 - + Atrophic gastritis O1 73 male none

5 - - Normal C0 67 female none

6 - - Superficial gastritis C0 67 female lansoprazole 15mg (due to gastritis)

7 - - Normal C0 62 male lansoprazole 15mg (due to esophagitis)

8 + - Superficial-atrophic gastritis O1 69 female none

9 + - Atrophic gastritis O1 76 male none

10 - + Atrophic gastritis C1 68 male lansoprazole 15mg (due to gastritis)

11 - - Normal C0 38 female none

12 - - Normal C0 54 female none

13 - + Atrophic gastritis C2 62 female none

14 - + Atrophic gastritis C3 49 female none

15 - - Normal C0 48 female none

16 + - Atrophic gastritis C3 52 female none

17 + - Superficial-atrophic gastritis O2 61 female none

18 - - Normal C0 63 male none

19 - - Normal C0 39 male none

20 - - Superficial gastritis C0 65 male none

21 + - Atrophic gastritis O2 63 female none

22 + - Atrophic gastritis C2 70 female none

23 - - Superficial gastritis C0 71 female none

24 + - Superficial gastritis C0 33 male none

25 - + Normal C0 72 female none

26 - + Atrophic gastritis C2 64 female lansoprazole 15mg (due to gastritis)

27 + - Atrophic gastritis O2 62 female none

28 - + Superficial-atrophic gastritis C2 46 female none

29 - - Normal C0 56 male none

30 - + Superficial-atrophic gastritis C3 70 male none

31 + - Superficial-atrophic gastritis O2 65 female none

32 + - Superficial-atrophic gastritis O2 48 male none

33 - + Superficial-atrophic gastritis C2 42 male none

34 + - Normal C0 57 female none

35 - - Normal C0 41 male none

36 - - Normal C0 44 female none

37 - + Atrophic gastritis C2 47 male none

38 - - Superficial gastritis C0 54 female none

39 - + Atrophic gastritis C2 74 male lansoprazole 15mg (due to gastritis)

40 - - Superficial gastritis C0 69 male none

41 + - Normal C0 40 female none

42 - + Atrophic gastritis C2 64 male none

43 - + Normal C0 58 male none

44 - - Superficial gastritis C0 70 female none

45 - + Superficial-atrophic gastritis C2 68 female none

46 + - Superficial-atrophic gastritis C2 67 male none

47 - + Atrophic gastritis C3 55 female none

Kimura–Takemoto classification of endoscopic atrophy^41^: C0 non-atrophy, C1 atrophy is limited to the antrum, C2 atrophy is limited to the minor area of the lesser curvature of the body, C3 atrophy exists in the major area of the lesser curvature of the body but does not extend beyond the cardia, O1 atrophy extends to the fundus over the cardia. Atrophic border of the body lies between the lesser curvature and anterior wall, O2 atrophic border of the body lies on the anterior wall, O3 atrophy is widespread with the border between the anterior wall and greater curvature.

^1^ Welch’s *t*-test showed no significant difference in ages between the *H. pylori-*negative and -positive groups (*p* = 0.191, α=.05).

^2^ Fisher’s exact test (two-tailed, confidence intervals at 95%) showed no significant difference in gender proportions between the *H. pylori*-negative and -positive groups (*p* = 0.348, α = 0.05).

^3^ Fisher’s exact test (two-tailed, confidence intervals at 95%) showed no significant difference in the proportions of individuals taking acid inhibitors between the *H. pylori*-negative and -positive groups (*p* = 0.167, α = 0.05).
