# Supplementary material for: Impact of Helicobacter pylori infection on fluid duodenal microbial community structure and microbial metabolic pathways
Source: BMC Microbiol. 2022 Jan 15;22:27. doi: 10.1186/s12866-022-02437-w (PMC8760755; doi:10.1186/s12866-022-02437-w)
Supplement: Supplementary file 2 — Additional file 2. [file 12866_2022_2437_MOESM2_ESM.docx]

**Supplementary Information 2: Relative mean abundance of phylum in the descending part of the duodenum**

% abundance (± SD)

Phylum *H. pylori* *H. pylori* *p*-value

negative (n=34) positive (n=13)

*Firmicutes* 40.24±15.91 34.77±11.28 ns

*Bacteroidetes* 21.35±13.81 17.69±11.07 ns

***Proteobacteria*  11.32±10.12 23.00±10.01 < 0.01**

***Actinobacteria* 9.53±7.10 5.08±2.63 < 0.01**

*Fusobacteria*  8.59±6.97 10.31±10.49 ns

N/A 4.58±7.24 5.67±12.45 ns

***TM7* 3.59±4.06 1.49±1.35 < 0.05**

*Tenericutes* 0.08±0.18 0.97±3.32 ns

*SR1* 0.33±0.80 0.40±0.84 ns

*Spirochaetes*  0.27±0.52 0.14±0.29 ns

*Synergistetes* 0.07±0.13 0.08±0.09 ns

*Cyanobacteria* 0.02±0.07 0.08±0.25 ns

{Unknown} 0.01±0.02 0.08±0.27 ns

{Unknown Phylum} *Bacteria-1*  0.01±0.02 0.03±0.04 ns

*GN02*  0.01±0.03 0.03±0.06 ns

*Chloroflexi* 0.01±0.02 0.00±0.01 ns

*OD1* 0.00±0.01 0.00±0.01 ns

*Acidobacteria* 0.00±0.03 0 *N*

*Euryarchaeota* 0 0 null

*Planctomycetes* 0.00±0.01 0 *N*

[*Thermi*] 0 0 null

{Unknown Phylum} *Bacteria-2* 0 0 null

*Chlamydiae* 0 0 null

Welch’s *t*-test was used to compare relative mean percent abundance of phyla between the *Helicobacter pylori* negative and positive groups. ns: nonspecific, *N*: existing only in the *H. pylori* negative group, null: the statistical significance test was invalid.
