# Supplementary material for: Impact of Helicobacter pylori infection on fluid duodenal microbial community structure and microbial metabolic pathways
Source: BMC Microbiol. 2022 Jan 15;22:27. doi: 10.1186/s12866-022-02437-w (PMC8760755; doi:10.1186/s12866-022-02437-w)
Supplement: Supplementary file 3 — Additional file 3. [file 12866_2022_2437_MOESM3_ESM.docx]

**Supplementary Information 3: Difference in the relative mean abundance of taxa at genus level with and without *Helicobacter pylori* infection.**

%abundance (±SD)

Genus *H. pylori* *H. pylori* *p* value

negative (n=34) positive (n=13)

*Streptococcus* 19.68±14.75 17.46±11.13 ns

*Prevotella* 15.62±10.10 10.85±7.29 ns

*Veillonella* 10.06±5.67 8.31±4.35 ns

*Fusobacterium* 5.90±5.88 8.90±9.64 ns

***Neisseria*  4.76±5.93 11.74±7.10 < 0.01**

***Rothia* 6.83±7.44 1.81±1.36 < 0.001**

N/A 4.46±7.28 5.67±12.45 ns

*Porphyromonas* 3.72±4.29 3.15±4.17 ns

[*Prevotella*] 2.69±3.96 2.48±3.38 ns

**{Unknown Order} TM7-3 2.74±3.54 0.80±0.89 < 0.01**

*Haemophilus* 1.68±2.19 3.79±4.43 ns

***Leptotrichia* 2.06±1.81 1.18±1.02 < 0.05**

*Actinomyces*  1.57±1.71 1.47±1.27 ns

*Actinobacillus* 1.32±3.11 2.07±2.53 ns

*Granulicatella* 1.32±1.37 1.32±1.55 ns

*Atopobium* 0.94±0.96 1.06±1.08 ns

{Unknown Genus} *Gemellaceae-2* 0.80±0.84 1.00±0.95 ns

*Helicobacter*  0 2.79±6.82 *P*

*Oribacterium*  0.92±0.90 0.53±0.54 ns

*Bulleidia* 0.72±0.61 0.67±0.31 ns

*Campylobacter* 0.66±1.01 0.63±0.53 ns

**{Unknown Genus} *Lachnospiraceae* 0.90±0.84 0.33±0.30 < 0.01**

***Megasphaera*** 0.72±0.76 0.38±0.33 **< 0.05**

*Anaerobacillus* 0.58±1.38 0.79±2.04 ns

{Unknown Genus} *Clostridiaceae*  0.43±0.57 0.44±0.52 ns

{Unknown Family} *Clostridiales-2*  0.41±0.54 0.45±0.81 ns

**{Unknown Genus} *F16*** **0.49±0.60** **0.21±0.23** **< 0.05**

*Selenomonas* 0.41±0.45 0.28±0.25 ns

{Unknown Family} *CW040*  0.36±0.60 0.31±0.58 ns

*Capnocytophaga* 0.29±0.50 0.47±0.81 ns

*Parvimonas*  0.36±0.61 0.35±0.36 ns

{Unknown Class} *SR1*  0.34±0.80 0.40±0.84 ns

*Treponema*  0.30±0.52 0.14±0.29 ns

{Unknown Genus} *Mycoplasmataceae* 0.00±0.00 0.92±3.33 ns

*Bacteroides* 0.33±0.89 0.40±1.12 ns

***Moryella*** 0.32±0.39 0.11±0.13 **< 0.05**

***Filifactor*** 0.28±0.45 0.09±0.16 **< 0.05**

{Unknown Genus} *Neisseriaceae*  0.17±0.25 0.34±0.30 ns

{Unknown Genus} [*Mogibacteriaceae*] 0.23±0.24 0.15±0.17 ns

*Delftia*  0.27±0.57 0.23±0.58 ns

*Dialister* 0.20±0.21 0.17±0.26 ns

*Tannerella*  0.12±0.16 0.22±0.32 ns

*Aggregatibacte*r 0.10±0.21 0.23±0.29 ns

*Lautropia*  0.10±0.24 0.15±0.25 ns

*Blautia* 0.17±0.39 0.08±0.28 ns

*Bifidobacterium* 0.17±0.27 0.18±0.55 ns

*Gemella* 0.09±0.12 0.22±0.20 ns

*Catonella*  0.14±0.17 0.08±0.09 ns

*Corynebacterium* 0.12±0.18 0.11±0.18 ns

{Unknown Genus} *Ruminococcaceae-2*  0.08±0.24 0.39±1.12 ns

*Lactobacillus*  0.14±0.53 0.02±0.04 ns

{Unknown Genus} Bacillaceae 0.10±0.21 0.13±0.31 ns

*Collinsella* 0.10±0.24 0.11±0.29 ns

*Ochrobactrum* 0.12±0.37 0.10±0.28 ns

*Butyrivibrio*  0.09±0.15 0.06±0.06 ns

{Unknown Genus} *Peptostreptococcaceae* 0.08±0.21 0.07±0.12 ns

{Unknown Family} *Bacteroidales* 0.07±0.20 0.03±0.07 ns

*TG5* 0.07±0.13 0.08±0.09 ns

{Unknown Genus} [*Weeksellaceae*] 0.05±0.06 0.13±0.15 ns

[*Ruminococcus*] 0.11±0.36 0.03±0.10 ns

{Unknown Family} *Actinomycetales* 0.01±0.02 0.15±0.55 ns

*Mycoplasma* 0.07±0.17 0.04±0.06 ns

*Eikenella*  0.04±0.08 0.10±0.12 ns

*Ralstonia* 0.06±0.18 0.00±0.00 ns

{Unknown Genus} *Aerococcaceae*  0.03±0.05 0.08±0.17 ns

*Pseudomonas* 0.03±0.09 0.08±0.13 ns

{Unknown Genus} Veillonellaceae 0.05±0.15 0.02±0.05 ns

*Agrobacterium* 0.06±0.17 0.00±0.01 ns

*Acinetobacter* 0.06±0.13 0.05±0.16 ns

*Sphingomonas* 0.02±0.05 0.07±0.15 ns

*Propionibacterium*  0.04±0.07 0.07±0.16 ns

*Bacillus* 0.04±0.08 0.07±0.18 ns

{Unknown Genus} *Erythrobacteraceae* 0.01±0.04 0.13±0.33 ns

*Staphylococcus*  0.05±0.17 0.03±0.08 ns

{Unknown Genus} *Leptotrichiaceae* 0 0.11±0.24 *P*

{Unknown Genus} *Enterobacteriaceae* 0.02±0.05 0.07±0.14 ns

{Unknown Genus} *Pseudomonadaceae*  0.03±0.12 0.02±0.02 ns

*Cardiobacterium* 0.04±0.13 0.02±0.03 ns

*Lachnospira* 0.06±0.20 0.00±0.01 ns

***Paludibacter* 0.04±0.06 0.01±0.02 < 0.05**

*Peptostreptococcus*  0 0.08±0.28 *P*

*Peptococcus*  0.03±0.04 0.03±0.08 ns

*Lysinibacillus*  0.03±0.09 0.03±0.07 ns

*Variovorax* 0.06±0.34 0.00±0.01 ns

*Coprococcus*  0.03±0.08 0.04±0.12 ns

*Rhodococcus* 0.03±0.09 0.02±0.05 ns

{Unknown Family} *Bacillales* 0.03±0.06 0.03±0.09 ns

{Unknown Genus} *Streptococcaceae*  0.02±0.03 0.02±0.02 ns

*Anaerostipes*  0.01±0.06 0.08±0.28 ns

*Mesorhizobium* 0.02±0.04 0.08±0.27 ns

*Methylobacterium* 0.03±0.09 0.01±0.03 ns

{Unknown Genus} *Planococcaceae* 0.02±0.06 0.03±0.11 ns

{Unknown Genus} *Erysipelotrichaceae* 0.00±0.01 0.10±0.28 ns

{Unknown} 0.01±0.02 0.08±0.27 ns

{Unknown Genus} *Rs-045*  0.02±0.03 0.01±0.01 ns

*Enhydrobacter* 0.01±0.02 0.05±0.10 ns

*Faecalibacterium*  0.03±0.08 0.00±0.00 ns

*Rhizobium* 0.02±0.09 0.00±0.00 ns

{Unknown Genus} *Acetobacteraceae*  0.02±0.09 0.00±0.01 ns

{Unknown Phylum} *Bacteria-1*  0.01±0.02 0.03±0.04 ns

*Schwartzia* 0.02±0.04 0.01±0.02 ns

*Klebsiella*  0.00±0.01 0.04±0.11 ns

{Unknown Family} *Rhodospirillal*es 0.03±0.17 0 *N*

*Slackia*  0.02±0.05 0.00±0.01 ns

*SMB53* 0.02±0.12 0 *N*

{Unknown Family} *RF39*  0.01±0.06 0.01±0.02 ns

*Sutterella* 0.03±0.15 0 *N*

{Unknown Genus} *Comamonadaceae* 0.01±0.03 0.01±0.02 ns

*Moraxella*  0 0.05±0.16 *P*

*Herbaspirillum*  0.02±0.09 0.02±0.06 ns

{Unknown Genus} [*Acidaminobacteraceae*] 0.01±0.03 0.01±0.01 ns

{Unknown Genus} *Moraxellaceae* 0.01±0.06 0.00±0.01 ns

{Unknown Genus} *Rhizobiaceae*  0.01±0.08 0.00±0.01 ns

{Unknown Genus} *Sphingomonadaceae*  0.01±0.05 0.02±0.04 ns

*Stenotrophomonas*  0.01±0.04 0.02±0.06 ns

*Mogibacterium*  0.01±0.02 0.01±0.02 ns

{Unknown Order} *BD1-5*  0.00±0.01 0.03±0.06 ns

*Kocuria* 0.02±0.07 0.01±0.04 ns

{Unknown Genus} *Coriobacteriaceae* 0.01±0.02 0.01±0.01 ns

*Lactococcus* 0.01±0.03 0.01±0.03 ns

*Kingella* 0.01±0.03 0.00±0.01 ns

{Unknown Genus} *Xenococcaceae*  0 0.07±0.26 *P*

*Alloscardovia*  0.01±0.05 0 *N*

{Unknown Genus} *Pasteurellaceae-2*  0.01±0.02 0.01±0.01 ns

{Unknown Genus} [*Tissierellaceae*] 0.01±0.04 0.00±0.01 ns

*Proteus* 0.02±0.11 0 *N*

*Parabacteroides*  0.01±0.04 0.03±0.11 ns

{Unknown Genus} *Caulobacteraceae*  0.01±0.04 0.00±0.01 ns

{Unknown Genus} *Actinomycetaceae*  0.01±0.02 0 *N*

{Unknown Family} *Streptophyta*  0.01±0.06 0.01±0.01 ns

*Halomonas* 0.00±0.01 0.03±0.06 ns

*SHD-231*  0.01±0.02 0.00±0.01 ns

*Dorea*  0.01±0.07 0 *N*

*Scardovia*  0.01±0.04 0 *N*

{Unknown Genus} *mitochondria*  0.01±0.07 0 *N*

*Flavobacterium*  0.01±0.04 0 *N*

*Sharpea*  0.01±0.05 0 *N*

*Anaerovorax* 0.01±0.03 0.01±0.02 ns

{Unknown Genus} *S24-7*  0.01±0.04 0 *N*

*Paracoccus*  0.01±0.04 0 *N*

*Burkholderia*  0.01±0.03 0 *N*

*Sneathia*  0 0.02±0.07 *P*

*Pyramidobacte*r 0.01±0.02 0 *N*

*Schlegelella* 0.01±0.02 0 *N*

*Peptoniphilus*  0.01±0.03 0 *N*

{Unknown Family} *Rhizobiales*  0.01±0.03 0.00±0.01 ns

*Enterococcus* 0 0.03±0.12 *P*

*Blvii28*  0.00±0.01 0.00±0.01 ns

{Unknown Order} *ZB2*  0.00±0.01 0.00±0.01 ns

{Unknown Genus} *Bradyrhizobiaceae* 0.01±0.04 0 *N*

{Unknown Order} *ML635J-21*  0.01±0.03 0 *N*

*Sediminibacterium*  0.00±0.03 0 *N*

*Chryseobacterium* 0.00±0.02 0 *N*

*Mobiluncus* 0.01±0.03 0.00±0.01 ns

*Bradyrhizobium* 0.01±0.03 0 *N*

{Unknown Genus} [*Barnesiellaceae*] 0.00±0.02 0 *N*

*Alloiococcus* 0.00±0.01 0 *N*

{Unknown Family} *MLE1-12* 0.00±0.02 0 *N*

*Paenibacillus*  0.00±0.02 0 *N*

{Unknown Genus} *Acidobacteriaceae* 0.00±0.03 0 *N*

{Unknown Genus} [*Paraprevotellaceae*] 0.00±0.01 0 *N*

*Acetobacter*  0.01±0.03 0. *N*

{Unknown Genus} *Phyllobacteriaceae* 0.00±0.02 0 *N*

{Unknown Family} *Rickettsiales* 0.00±0.02 0.00±0.01 ns

*Phyllobacterium* 0.00±0.01 0 *N*

*Bdellovibrio* 0.00±0.01 0 *N*

*Mycobacterium*  0.00±0.02 0 *N*

{Unknown Genus} *Trebouxiophyceae* 0.00±0.02 0 *N*

*Hydrogenophilus* 0.00±0.01 0 *N*

*Microbacterium* 0.00±0.01 0 *N*

{Unknown Genus} *Sporichthyaceae* 0.00±0.01 0 *N*

*Bilophila*  0.00±0.01 0 *N*

{Unknown Genus} *Xanthomonadaceae* 0.00±0.01 0 *N*

*Oscillospira*  0.00±0.02 0 *N*

*Ruminococcus*  0 0.01±0.03 *P*

{Unknown Genus} *Pasteurellaceae-1* 0 0.00±0.01 *P*

{Unknown Genus} *Pseudonocardiaceae*  0.00±0.02 0 *N*

*Wautersiella*  0.00±0.01 0 *N*

[*Eubacterium*] 0.00±0.01 0.00±0.01 ns

*Leucobacter*  0.00±0.01 0 *N*

*Desulfobulbus*  0.00±0.01 0 *N*

*Caulobacter*  0.00±0.01 0 *N*

{Unknown Genus} *Oxalobacteraceae*  0 0.01±0.02 *P*

*Rheinheimera* 0.00±0.02 0 *N*

{Unknown Family} *DHVE3* 0 0 null

{Unknown Genus} *Nocardioidaceae*  0.00±0.01 0 *N*

{Unknown Genus} *Propionibacteriaceae*  0 0 null

{Unknown Genus} *Chitinophagaceae* 0.00±0.02 0 *N*

*Pseudoramibacter_Eubacterium* 0 0 null

*Acidaminococcus* 0 0.00±0.01 *P*

*Janthinobacterium* 0.00±0.01 0 *N*

*Erwinia* 0.00±0.01 0 *N*

{Unknown Class} *Bacteroidetes-2* 0.00±0.01 0 *N*

{Unknown Family} *Clostridiales-1* 0 0 null

{Unknown Family} *Burkholderiales* 0 0.00±0.01 *P*

*Desulfovibrio*  0 0 null

*Roseburia* 0.00±0.01 0 *N*

*Phascolarctobacterium*  0.00±0.01 0 *N*

{Unknown Genus} *Sinobacteraceae* 0.00±0.01 0 *N*

*Sphaerochaeta* 0 0 null

*Thermicanus* 0 0 null

*Finegoldia* 0.00±0.01 0 *N*

*Bosea*  0 0.00±0.01 *P*

{Unknown Genus} BS11 0 0.00±0.01 *P*

*Shewanella*  0.00±0.01 0 *N*

*Acholeplasma* 0.00±0.01 0 *N*

*Cloacibacterium* 0.00±0.01 0 *N*

{Unknown Genus} *Isosphaeraceae* 0.00±0.01 0 *N*

*Rhodoplanes*  0.00±0.01 0 *N*

*Serratia* 0 0 null

*Odoribacter* 0 0 null

{Unknown Genus} *Flavobacteriaceae* 0 0 null

{Unknown Order} *Alphaproteobacteria*  0 0.00±0.01 *P*

{Unknown Genus} *Legionellaceae* 0 0 null

*Deinococcus*  0 0 null

{Unknown Genus} *Micrococcaceae* 0 0 null

*Jiangella* 0 0 null

{Unknown Class} *Bacteroidetes-1*  0 0 null

{Unknown Genus} *p-2534-18B5* 0 0 null

*Crocinitomix*  0 0 null

*Pediococcus*  0 0 null

*Novosphingobium*  0 0 null

{Unknown Genus} *Desulfobacteraceae* 0 0 null

{Unknown Genus} *Aeromonadaceae*  0 0 null

{Unknown Family} *iii1-15*  0 0 null

{Unknown Genus} *C111*  0 0 null

*Nesterenkonia* 0 0 null

{Unknown Class} *OD1* 0 0 null

*Anaerospora*  0 0 null

{Unknown Genus} *Dethiosulfovibrionaceae* 0 0 null

{Unknown Phylum} Bacteria-2 0 0 null

*Candidatus Solibacter* 0 0 null

*Yonghaparkia* 0 0 null

*Saccharopolyspora* 0 0 null

*Lewinella* 0 0 null

{Unknown Order} *BME43* 0 0 null

*Adhaeribacter* 0 0 null

{Unknown Order} *VC2_1_Bac22*  0 0 null

*Candidatus Rhabdochlamydia* 0 0 null

{Unknown Family} *mle1-48*  0 0 null

{Unknown Family} *Euglenozoa*  0 0 null

{Unknown Genus} *Gemellaceae-1* 0 0 null

*Carnobacterium*  0 0 null

*ph2* 0 0 null

{Unknown Genus} *Ruminococcaceae-1*  0 0 null

{Unknown Genus} *Pirellulaceae*  0 0 null

*Phenylobacterium* 0 0 null

*Devosia* 0 0 null

*Acidovorax* 0 0 null

{Unknown Genus} *Bacteriovoracaceae* 0 0 null

{Unknown Genus} *Desulfarculaceae*  0 0 null

{Unknown Family} *Thiohalorhabdales* 0 0 null

{Unknown Family} *ML615J-28* 0 0 null

Welch’s *t*-test was used to compare relative mean percent abundance of each genus between the *H. pylori* negative and positive groups. ns: nonspecific, *N*: existing only in the *H. pylori* negative group, *P*: existing only in the *H. pylori* positive group, null: the statistical significance test was invalid.
