# Supplementary material for: Impact of Helicobacter pylori infection on fluid duodenal microbial community structure and microbial metabolic pathways
Source: BMC Microbiol. 2022 Jan 15;22:27. doi: 10.1186/s12866-022-02437-w (PMC8760755; doi:10.1186/s12866-022-02437-w)
Supplement: Supplementary file 4 — Additional file 4. [file 12866_2022_2437_MOESM4_ESM.docx]

**Supplementary Information 4: Difference in the KEGG metabolic pathways with and without *Helicobacter pylori* infection.**

KEGG ko-abundance

Class pathway Pathway name *H. pylori* negative (n=34) *H. pylori* positive (n=13)

(ko number) 1^st^. quartile Median 3^rd^. quartile 1^st^. quartile Median 3^rd^. quartile p value

M ko00010 Glycolysis 41633612.1 54217471.7 61501257.7 43261735.3 55710950.0 57050698.8 ns

M ko00020 Citrate cycle (TCA cycle) 21096374.9 24982754.2 30481339.6 19230742.0 28003654.2 29538226.4 ns

M ko00030 Pentose phosphate pathway 26645757.4 30839809.1 35395330.4 28444067.6 34074469.0 36839085.1 ns

M ko00040 Pentose and glucuronate 6238980.5 8078933.1 9104703.2 7182925.3 8100606.8 9074922.6 ns

interconversions

M ko00051 Fructose and mannose metabolism 28328190.5 36891370.0 45764370.8 24920716.7 33861596.1 42756175.3 ns

M ko00052 Galactose metabolism 24178762.2 33415813.1 39180723.4 22911964.9 30506426.5 34071520.1 ns

M ko00053 Ascorbate and aldarate metabolism 3357227.8 4722741.7 5968408.8 4530735.8 4854066.9 6744004.0 ns

M ko00061 Fatty acid biosynthesis 21014491.0 25508985.0 27708537.6 23152580.0 26382884.1 29472405.3 ns

M ko00062 Fatty acid elongation 0.0 0.0 0.0 0.0 0.0 0.0 null

M ko00071 Fatty acid degradation 6194779.2 7102332.9 7792761.7 6740719.8 7253510.2 8227517.7 ns

M **ko00072 Synthesis and degradation of** 1126877.1 **1335885.8** 1819174.7 1353173.5 **1823878.4** 2817567.0 **< 0.05**

**ketone bodies**

M ko00100 Steroid biosynthesis 92.9 431.8 1415.1 0.0 233.2 1323.2 ns

M ko00120 Primary bile acid biosynthesis 12586.1 34335.0 77866.5 20590.1 26548.3 35778.8 ns

M ko00121 Secondary bile acid biosynthesis 34328.4 52008.4 103619.7 50879.4 83098.3 144478.5 ns

M ko00130 Ubiquinone and other 7775851.3 9328524.5 11793368.5 5446099.8 10689934.5 11606368.8 ns

terpenoid-quinone biosynthesis

M ko00140 Steroid hormone biosynthesis 71953.3 138620.3 234668.6 51255.3 99895.1 148101.0 ns

M ko00190 Oxidative phosphorylation 41445240.3 48659694.2 54638098.2 50533141.4 54358400.2 58106925.1 ns

M ko00195 Photosynthesis 16151790.9 19052631.5 21892738.9 16168203.0 20338112.2 21119467.3 ns

M ko00220 Arginine biosynthesis 12979792.8 16510492.0 18496490.4 15668837.4 17222801.8 20296205.4 ns

M ko00230 Purine metabolism 92655330.8 113981810.6 126877357.6 95001563.8 117836553.3 122914183.3 ns

M ko00232 Caffeine metabolism 0.0 430.5 2564.6 0.0 122.2 1216.0 ns

M ko00240 Pyrimidine metabolism 75975692.3 93978254.3 103794466.2 78127466.4 96422201.6 101248385.7 ns

M ko00250 Alanine, aspartate and glutamate 30844948.1 37447548.3 40664091.4 36286001.7 39591693.1 42848091.8 ns

metabolism

M ko00253 Tetracycline biosynthesis 0.0 0.0 0.0 0.0 0.0 0.0 null

M ko00260 Glycine, serine and threonine 31671499.6 39466964.9 43676398.7 37926025.0 40731045.2 44193806.3 ns

metabolism

M ko00261 Monobactam biosynthesis 8230865.3 9781344.1 11105404.4 9574455.9 10354728.4 11197892.6 ns

M ko00270 Cysteine and methionine metabolism 38840585.6 47108777.1 52689923.8 46130211.1 49624642.1 54502038.8 ns

M ko00280 Valine, leucine and isoleucine 7137849.7 8243999.2 9857581.0 8671644.2 9244987.3 10547069.4 ns

degradation

M ko00281 Geraniol degradation 40368.9 312911.5 538946.3 170604.9 211020.1 772015.9 ns

M ko00290 Valine, leucine and isoleucine 11945167.7 15342681.0 18990524.4 13536079.0 16341291.9 19658435.2 ns

biosynthesis

M ko00300 Lysine biosynthesis 21573793.2 27050237.8 30831394.7 23508304.8 26084476.5 30552784.3 ns

M ko00310 Lysine degradation 3908170.6 4691565.1 6250851.9 4577895.5 5845179.7 7633707.9 ns

M ko00311 Penicillin and cephalosporin 655682.5 847784.8 1170121.8 854039.7 969496.7 1343962.4 ns

biosynthesis

M ko00330 Arginine and proline metabolism 10326214.3 12198039.4 13987133.7 12654951.3 14290882.2 14973160.1 ns

M ko00332 Carbapenem biosynthesis 1188566.7 1898365.3 2358939.3 1726080.2 2231949.7 2479531.7 ns

M ko00333 Prodigiosin biosynthesis 5153729.5 6466270.3 7076955.9 5795165.3 6791275.3 7496624.9 ns

M ko00340 Histidine metabolism 10028809.5 12157739.4 13056994.9 11208516.0 14000051.2 14684988.2 ns

M ko00350 Tyrosine metabolism 28796.8 7027097.4 7752907.9 7182685.9 8370522.3 8648983.7 ns

M ko00360 Phenylalanine metabolism 5060671.4 5670459.4 7006467.8 6377422.1 6587663.0 8473349.3 ns

M ko00361 Chlorocyclohexane and chlorobenzene 125938.8 252888.0 508588.3 114557.9 162955.4 344148.6 ns

degradation

M ko00362 Benzoate degradation 2405411.2 3177123.5 4029701.3 2998345.5 3729221.8 4648023.1 ns

M ko00363 Bisphenol degradation 0.0 124.4 1248.4 143.7 596.7 1562.8 null

M ko00364 Fluorobenzoate degradation 12016.3 54241.5 15639.4 10871.5 27355.6 72004.9 ns

M ko00365 Furfural degradation 0.0 0.0 1986.2 0.0 122.2 1149.3 null

M **ko00380** **Tryptophan metabolism** 1511250.1 **2254680.8** 2828070.2 2038660.5 **2759405.6** 4675930.6 **< 0.05**

M ko00400 Phenylalanine, tyrosine and tryptophan 27283577.7 33066988.8 39531510.3 32013253.6 34059206.9 39083190.2 ns

biosynthesis

M ko00401 Novobiocin biosynthesis 4204801.7 4889910.2 5598086.0 4505569.1 5662949.1 5989182.1 ns

M ko00404 Staurosporine biosynthesis 0.0 1658.6 8559.0 35.9 1786.2 25001.3 null

M ko00405 Phenazine biosynthesis 1960816.3 2615042.0 3092113.6 2311554.7 2788282.5 3175337.7 ns

M ko00410 beta-Alanine metabolism 3031847.0 3691762.4 4658121.4 2933841.7 4247395.6 4924333.6 ns

M ko00430 Taurine and hypotaurine metabolism 5131769.4 6202076.3 6647057.0 6150083.1 7010053.8 7593125.1 ns

M ko00440 Phosphonate and phosphinate 1139827.5 1394862.7 1884257.3 715503.6 1496614.7 1678174.1 ns

metabolism

M ko00450 Selenocompound metabolism 12422171.5 14395645.9 16729218.3 13534753.1 15524780.3 17503466.3 ns

M ko00460 Cyanoamino acid metabolism 5374904.1 6495714.8 7296560.2 5758235.0 6958452.6 7435625.4 ns

M ko00471 D-Glutamine and D-glutamate 6787416.3 8080394.2 8831741.4 6951399.7 8066602.7 8951783.7 ns

metabolism

M ko00472 D-Arginine and D-ornithine metabolism 413739.2 648575.4 1198200.1 802795.3 843356.2 1054469.9 ns

M ko00473 D-Alanine metabolism 4974004.6 6089430.8 7304867.3 4956020.8 6837864.7 7594743.0 ns

M ko00480 Glutathione metabolism 10117449.1 12303721.2 13446687.4 11851352.6 12527586.1 13761984.1 ns

M ko00500 Starch and sucrose metabolism 32329164.5 44083011.3 56165635.1 29620801.8 46667361.4 50148130.8 ns

M **ko00510** **N-Glycan biosynthesis** 646002.5 **907554.4** 1359978.7 297990.6 **700748.0** 919158.8 **< 0.05**

M ko00511 Other glycan degradation 8545326.2 10513399.7 16544004.9 5007916.8 11276258.9 13881566.5 ns

M ko00513 Various types of N-glycan biosynthesis 1558304.9 2123403.6 3496256.8 839131.9 2299323.6 2870744.1 ns

M ko00514 Other types of O-glycan biosynthesis 91460.7 165741.8 408942.8 78772.8 154825.7 174728.7 ns

M ko00515 Mannose type O-glycan biosynthesis 91460.7 164974.3 408504.1 77942.8 154825.7 174728.7 ns

M ko00520 Amino sugar and nucleotide sugar 49640362.5 62281498.1 70486244.0 44893646.6 64297992.0 72940929.5

metabolism

M ko00521 Streptomycin biosynthesis 11569094.3 13772822.0 16239226.0 13093725.4 14879784.8 16326667.6 ns

M ko00523 Polyketide sugar unit biosynthesis 7121892.1 8314915.6 9744053.3 7483547.1 8006398.0 9435367.8 ns

M ko00524 Neomycin, kanamycin and gentamicin 1672620.6 1976413.9 2634570.3 1666427.1 2436921.6 2820044.6 ns

biosynthesis

M ko00525 Acarbose and validamycin biosynthesis 3542671.5 4164224.5 4849304.0 3752847.1 4128403.0 4793320.6 ns

M ko00531 Glycosaminoglycan degradation 2790208.6 3623037.1 5436479.5 1739067.6 4423911.6 4699965.3 ns

M ko00532 Glycosaminoglycan biosynthesis 0.0 0.0 0.0 0.0 0.0 0.0 null

M ko00534 Glycosaminoglycan biosynthesis- 0.0 0.0 0.0 0.0 0.0 0.0 null

heparan sulfate/heparin

M ko00540 Lipopolysaccharide biosynthesis 13447368.7 20921215.8 25652391.0 21266883.5 26477202.8 31900260.3 ns

M ko00550 Peptidoglycan biosynthesis 35291239.1 45692228.8 49169084.1 33242374.3 44896636.0 49941954.3 ns

M ko00561 Glycerolipid metabolism 10319977.6 13992440.1 16536050.8 11371809.6 16414039.5 17251622.9 ns

M ko00562 Inositol phosphate metabolism 3455493.5 4319903.9 4783256.8 3994332.6 4747087.1 5121875.2 ns

M ko00563 Glycosylphosphatidylinositol (GPI)- 0.0 665.0 3612.5 499.5 1955.0 4560.0

anchor biosynthesis

M ko00564 Glycerophospholipid metabolism 18737876.9 22356353.3 24349216.0 21985575.9 24992369.0 27019346.3 ns

M **ko00565** **Ether lipid metabolism** 78421.4 **150966.1** 178307.8 294643.9 **394815.2** 494657.0 **< 0.001**

M **ko00571** **Lipoarabinomannan (LAM)** 203496.6 **397199.6** 861599.4 171964.3 **191311.5** 370699.8 **< 0.05**

**biosynthesis**

M ko00572 Arabinogalactan biosynthesis- 754707.3 1131005.4 1771013.2 581689.5 912128.2 1414727.6 ns

Mycobacterium

M ko00590 Arachidonic acid metabolism 2218508.1 3002572.1 3861709.1 2500897.8 3520207.4 4187127.2 ns

M **ko00591** **Linoleic acid metabolism** 45615.1 **85148.3** 152804.4 234909.9 **389478.0** 493380.2 **< 0.001**

M **ko00592** **alpha-Linolenic acid metabolism** 98883.7 **132544.9** 179418.1 317677.7 **405656.4** 500286.1 **< 0.001**

M ko00600 Sphingolipid metabolism 5179866.9 6145526.8 9216650.7 3626969.6 6336044.7 7657656.9 ns

M ko00601 Glycosphingolipid biosynthesis-lacto 0.0 0.0 0.0 0.0 0.0 0.0 null

and neolacto series

M ko00603 Glycosphingolipid biosynthesis-globo 2467061.3 3122220.6 5143973.0 1604399.3 3562515.3 4254909.9 ns

and isoglobo series

M ko00604 Glycosphingolipid biosynthesis-ganglio 1558304.9 2123403.6 3496256.8 839131.9 2299323.6 2870744.1 ns

series

M ko00620 Pyruvate metabolism 39784518.2 47013060.6 53898784.7 47533882.8 52722297.8 57760557.1 ns

M ko00621 Dioxin degradation 554679.3 832655.5 1140247.9 846941.9 1153652.9 1297329.8 ns

M ko00622 Xylene degradation 644925.3 1030485.0 1358234.3 1048887.0 1213701.3 1361048.9 ns

M ko00623 Toluene degradation 11644.1 57731.1 164332.7 11401.8 25131.9 65746.4 ns

M ko00624 Polycyclic aromatic hydrocarbon 11061.3 38960.7 141703.1 7744.0 24924.7 33763.1 ns

degradation

M ko00625 Chloroalkane and chloroalkene 1594685.4 2384537.2 3074640.7 1963428.6 3082104.0 3184591.4 ns

degradation

M ko00626 Naphthalene degradation 1125017.1 2052491.0 2789156.3 1798130.6 2038181.4 2805440.4 ns

M ko00627 Aminobenzoate degradation 1831561.0 2449784.3 3093731.2 2487033.3 2778533.3 3778239.1 ns

M ko00630 Glyoxylate and dicarboxylate 20984309.5 24979229.9 28373946.3 23142002.1 27421589.8 29785798.4 ns

metabolism

M ko00633 Nitrotoluene degradation 330882.3 497468.0 667257.2 624676.1 693312.2 861754.2 ns

M ko00640 Propanoate metabolism 20150479.8 24233111.1 27022679.4 24239639.6 26998882.1 29919627.9

M ko00642 Ethylbenzene degradation 4851.9 20538.4 44245.9 5641.8 15686.8 38434.6 ns

M ko00643 Styrene degradation 562519.7 853462.3 1162253.9 790331.0 932779.7 1744197.5 ns

M ko00650 Butanoate metabolism 20652280.4 24194255.6 25781224.4 22930774.4 26052713.2 27389271.2 ns

M ko00660 C5-Branched dibasic acid metabolism 7683961.5 10175406.0 11722800.1 8834586.0 9829260.7 11593769.0 ns

M ko00670 One carbon pool by folate 21792462.3 27885843.2 30480777.5 22390602.8 28285989.7 29427756.5 ns

M ko00680 Methane metabolism 22890752.8 28968832.3 31327119.5 26192838.5 30115433.2 31700701.4 ns

M ko00710 Carbon fixation in photosynthetic 21942952.9 26416619.3 29290673.4 22942730.6 27268696.2 30427048.4 ns

organisms

M ko00720 Carbon fixation pathways in prokaryotes 31066961.8 37602628.3 41633581.9 35587066.8 39693789.0 43648424.0 ns

M ko00730 Thiamine metabolism 17335375.4 20114735.5 21777909.3 19239175.3 20464323.1 23172615.3 ns

M ko00740 Riboflavin metabolism 8776945.0 10645813.5 12562452.4 10038280.8 12050000.6 12581342.7 ns

M ko00750 Vitamin B6 metabolism 6261007.8 7859233.8 9132855.2 6782169.5 8274279.3 9337236.4 ns

M ko00760 Nicotinate and nicotinamide metabolism 18137265.9 21992823.1 24236856.7 22076998.1 23120420.3 25691392.1 ns

M ko00770 Pantothenate and CoA biosynthesis 19779927.5 23454688.9 27042578.2 21777424.1 26225975.1 27968100.6 ns

M **ko00780** **Biotin metabolism** 12987027.7 **15103400.2** 17153600.8 14854424.6 **19564468.4** 20905606.2 **< 0.05**

M ko00785 Lipoic acid metabolism 1521938.0 1923874.4 2463867.2 1649328.1 1950558.0 2352231.7 ns

M ko00790 Folate biosynthesis 22219073.7 25567639.8 28707008.8 25624229.6 27059765.5 29010150.9 ns

M ko00791 Atrazine degradation 135937.1 313522.9 598162.3 165188.3 419830.0 728220.9 ns

M ko00830 Retinol metabolism 1672727.8 2137999.7 2763621.2 1574971.8 2178361.5 2625602.7 ns

M ko00860 Porphyrin and chlorophyll metabolism 20236480.0 23517081.3 27604611.0 23723727.5 25126262.4 32587134.5 ns

M ko00900 Terpenoid backbone biosynthesis 20062008.5 24351723.5 25923242.1 22180482.3 25446066.4 27763393.8 ns

M ko00901 Indole alkaloid biosynthesis 0.0 0.0 418.9 0.0 0.0 122.2 null

M ko00902 Monoterpenoid biosynthesis 0.0 0.0 0.0 0.0 53.2 457.5 null

M ko00903 Limonene and pinene degradation 45381.7 129092.9 360201.8 52325.6 72819.7 114722.0 ns

M **ko00906** **Carotenoid biosynthesis** 85617.6 **129310.3** 186276.4 145417.5 **510048.5** 622715.8 **< 0.01**

M ko00908 Zeatin biosynthesis 2475164.5 2979953.7 3604280.0 2370909.0 3158213.1 3334979.7 ns

M ko00909 Sesquiterpenoid and triterpenoid 2086.2 4924.0 16056.1 3475.0 4520.1 8661.6 ns

biosynthesis

M ko00910 Nitrogen metabolism 13637967.8 17260386.1 19052884.4 14040783.8 15918881.7 17609707.1 ns

M ko00920 Sulfur metabolism 12477984.6 14034247.7 16462535.1 13328244.2 16308314.3 18063362.1 ns

M ko00930 Caprolactam degradation 24929.4 105312.9 275959.6 32019.6 47456.5 106381.7 ns

M **ko00940** **Phenylpropanoid biosynthesis** 268476.5 **348411.2** 592736.6 478950.5 **598460.1** 1004215.6 **< 0.01**

M ko00941 Flavonoid biosynthesis 808.2 4534.0 10070.0 3984.6 5369.3 10124.7 ns

M ko00943 Isoflavonoid biosynthesis 0.0 0.0 0.0 0.0 0.0 0.0 null

M ko00944 Flavone and flavonol biosynthesis 589.8 973.8 6541.8 390.0 1463.3 1894.0 ns

M ko00945 Stilbenoid, diarylheptanoid and gingerol 808.2 4534.0 10070.0 3984.6 5369.3 10124.7 ns

biosynthesis

M ko00950 Isoquinoline alkaloid biosynthesis 1791440.9 2476452.4 3059222.3 2451435.0 3165711.3 3556322.3 ns

M ko00960 Tropane, piperidine and pyridine 3129086.7 3874561.3 4822167.5 3085475.3 4298922.9 4887972.8 ns

alkaloid biosynthesis

M ko00965 Betalain biosynthesis 562517.3 716131.9 1166209.5 590134.8 918780.4 1032144.7 ns

M ko00966 Glucosinolate biosynthesis 875689.2 1220356.2 1625953.4 991918.5 1322849.4 1597514.3 ns

M ko00970 Aminoacyl-tRNA biosynthesis 142610356.1 171520646.3 195005434.4 146053790.5 172094256.4 191278607.9 ns

M ko00980 Metabolism of xenobiotics by 813637.4 1269071.1 1753301.6 1182414.6 1578356.0 1780057.1 ns

cytochrome P450

M ko00981 Insect hormone biosynthesis 29034.4 54069.3 168915.6 31796.4 49385.6 67723.0 ns

M ko00982 Drug metabolism - cytochrome P450 819053.5 1269825.7 1754005.2 1185122.7 1583010.5 1790645.8 ns

M ko00983 Drug metabolism - other enzymes 13445735.6 17610183.4 19792190.4 13500365.3 17747001.9 18955972.0 ns

M ko00984 Steroid degradation 511.9 17781.2 60998.1 441.0 4083.9 12505.2 ns

M ko00999 Biosynthesis of various secondary 3021670.4 4033380.7 4624147.6 3440203.2 4317513.0 4618033.5 ns

metabolites - part 1

M ko01040 Biosynthesis of unsaturated fatty acids 3181753.8 4035188.5 4556308.8 3716134.8 4292665.8 4559957.2 ns

M ko01051 Biosynthesis of ansamycins 2940258.5 3418442.4 4345784.3 3143631.2 3463323.0 4047518.9 ns

M ko01052 Type I polyketide structures 0.0 0.0 0.0 0.0 0.0 0.0 null

M **ko01053** **Biosynthesis of siderophore group** 1227510.4 **2025414.2** 2657402.2 701941.6 **1127312.8** 1571755.9 **< 0.01**

**nonribosomal peptides**

M ko01054 Nonribosomal peptide structures 284427.6 391073.5 554421.0 361463.8 426226.9 527956.8 ns

M ko01055 Biosynthesis of vancomycin group 1786445.0 2099433.4 2444178.3 1929188.1 2081227.9 2428151.2 ns

antibiotics

M ko01056 Biosynthesis of type II polyketide 0.0 0.0 0.0 0.0 0.0 0.0 null

backbone

M ko01057 Biosynthesis of type II polyketide 0.0 0.0 0.0 0.0 0.0 0.0 null

products

M ko01059 Biosynthesis of enediyne antibiotics 0.0 96.6 778.0 0.0 166.7 380.0 null

M **ko01062** **Biosynthesis of terpenoids and** 50943.0 **92469.4** 158821.7 143659.6 **428251.7** 512619.3 **< 0.01**

**steroids**

M ko01100 Metabolic pathways 643267293.7 781784556.9 870563064.2 743996745.9 821733985.1 886430049.8 ns

M ko01110 Biosynthesis of secondary metabolites 274225958.3 328607950.5 378111903.5 323839239.8 347626256.9 380591472.6 ns

M ko01120 Microbial metabolism in diverse 158425902.0 181691058.5 207314304.0 184464933.7 197496811.8 212810656.4 ns

environments

M ko01130 Biosynthesis of antibiotics 212115458.9 258413849.5 295394119.1 242935436.0 272103511.0 292878839.1 ns

M ko01200 Carbon metabolism 97926624.5 116811645.0 126936751.1 114394586.4 119686357.3 131700451.2 ns

M ko01210 2-Oxocarboxylic acid metabolism 19174137.8 24387335.5 27486465.2 21732309.4 28073278.7 30295014.0 ns

M ko01212 Fatty acid metabolism 20779782.1 25482970.2 27071516.6 24342082.8 27538005.3 28782566.1 ns

M ko01220 Degradation of aromatic compounds 2480405.1 3810010.3 5171796.1 3994651.2 5084791.8 5574328.0 ns

M ko01230 Biosynthesis of amino acids 130527118.5 152843383. 1181162730.9 146447338.4 167674230.1 181349234.7 ns

H ko01501 beta-Lactam resistance 17387972.6 22911946.9 26021638.7 19139512.3 23295044.3 26588966.3 ns

H ko01502 Vancomycin resistance 11594969.4 14548812.0 15946630.4 11386180.0 15037129.4 16087954.8 ns

H ko01503 Cationic antimicrobial peptide (CAMP) 8781274.0 11104596.0 13089854.9 11350548.7 13829234.3 15063234.5 ns

resistance

H ko01523 Antifolate resistance 9191810.8 11874003.4 13459698.2 9516724.5 11453042.6 11937239.0 ns

H ko01524 Platinum drug resistance 1561918.4 1941523.9 2504580.3 1978005.3 2121699.4 2349469.1 ns

E ko02010 ABC transporters 89811280.9 115469128.6 129180013.4 100115016.4 127882423.1 136781818.9 ns

E ko02020 Two-component system 38578696.3 49581758.1 55106640.7 46744147.5 51623371.3 53456584.8 ns

C ko02024 Quorum sensing 59751969.3 77384159.0 87808637.6 59180828.6 78855048.0 83839314.4 ns

C ko02025 Biofilm formation – Pseudomonas 3509425.8 4080916.5 4983228.3 3248475.9 4466290.8 4655795.1 ns

aeruginosa

C ko02026 Biofilm formation - Escherichia coli 9611071.2 11640763.3 13155892.6 8941351.8 12123590.3 13413393.5 ns

C ko02030 Bacterial chemotaxis 2957642.8 3955426.2 4521341.6 3103753.5 3889637.3 6339460.9 ns

C ko02040 Flagellar assembly 1784432.2 2714017.8 4722753.1 1897723.4 3164258.0 8768672.3 ns

E ko02060 Phosphotransferase system (PTS) 19501969.1 29975131.1 42477905.7 22882153.7 27878143.2 37799121.1 ns

G ko03008 Ribosome biogenesis in eukaryotes 2484543.2 2983910.6 3564012.6 2355137.9 2972712.2 3309373.0 ns

G ko03010 Ribosome 124582407.0 149382224.8 166454064.5 127959893.7 156268957.6 163617954.0 ns

G ko03013 RNA transport 1888092.8 2350298.4 2761421.8 2214892.0 2517300.1 2908069.6 ns

G ko03018 RNA degradation 22446180.7 26678795.0 29927067.6 23523201.8 28038973.5 30545736.6 ns

G ko03020 RNA polymerase 7850070.7 9212416.6 11024057.5 7679830.2 10069483.6 10685169.1 ns

G ko03022 Basal transcription factors 4675.9 8613.3 24388.2 2109.0 9911.0 22280.0 ns

G ko03030 DNA replication 29406774.4 35848989.3 39895222.3 29802914.3 37809671.6 38975952.6 ns

G ko03050 Proteasome 95422.9 188036.5 433113.9 79052.8 91671.7 200332.2 < 0.05

G ko03060 Protein export 26606231.2 31083687.3 37256174.2 27612339.8 34879886.5 36509714.4 ns

E ko03070 Bacterial secretion system 24469093.0 27649866.1 31582318.4 26033828.2 31374614.2 32620552.7 ns

O ko03320 PPAR signaling pathway 4184922.3 5288788.5 5916308.2 3939190.6 4328448.0 5407202.2 ns

G ko03410 Base excision repair 17569915.0 20817145.5 24157491.1 18627228.4 22737762.1 24211757.4 ns

G ko03420 Nucleotide excision repair 17176575.5 19951853.8 22683160.9 16862388.7 20658164.2 23096990.8 ns

G ko03430 Mismatch repair 34486761.6 41757750.9 46937103.8 35871546.6 43097493.9 47624288.5 ns

G ko03440 Homologous recombination 41576117.9 49088925.7 56158600.5 43092732.6 51687046.5 57091672.0 ns

G ko03450 Non-homologous end-joining 6628.0 22839.1 65896.9 4491.2 12124.1 22239.4 ns

E ko04011 MAPK signaling pathway – yeast 244963.3 328689.2 372574.2 492668.5 579710.0 751629.7 < 0.001

E ko04013 MAPK signaling pathway - fly 994327.0 1549317.4 1801143.3 959695.2 1408877.7 1644318.7 ns

E ko04014 Ras signaling pathway 0.0 0.0 0.0 0.0 0.0 0.0 null

E ko04016 MAPK signaling pathway – plant 2194046.9 2991487.2 3447082.5 2530028.8 3699389.2 3895027.4 ns

E ko04024 cAMP signaling pathway 75.5 629.8 7239.3 0.0 111.0 565.0 null

E ko04064 NF-kappa B signaling pathway 0.0 0.0 0.0 0.0 0.0 0.0 null

E ko04066 HIF-1 signaling pathway 4984583.6 6595040.0 7985182.7 5094828.7 6973707.3 7114194.1 ns

E ko04068 FoxO signaling pathway 1389187.3 2072810.6 2700373.0 1482296.7 2259434.6 2467905.8 ns

E ko04070 Phosphatidylinositol signaling 2932954.2 3534542.0 3975924.7 3735850.7 4431842.4 5115342.1 < 0.01

system

E ko04071 Sphingolipid signaling pathway 52642.5 119307.0 277172.9 42744.7 60807.1 63408.7 < 0.05

E ko04072 Phospholipase D signaling pathway 148267.4 212611.7 330670.9 331885.1 607713.3 744998.9 < 0.001

E ko04080 Neuroactive ligand-receptor interaction 1227.6 5306.1 19717.0 1208.2 3890.2 7255.1 ns

C ko04112 Cell cycle – Caulobacter 21033598.0 25718134.3 28938016.4 22560439.8 26888742.7 29508721.2 ns

C ko04113 Meiosis – yeast 141023.8 313964.3 461434.1 97774.9 162463.6 223353.5 < 0.05

C ko04115 p53 signaling pathway 42032.1 67277.6 131841.9 24515.8 83762.7 131778.1 ns

G ko04122 Sulfur relay system 7343402.2 8760290.3 11258540.2 7370285.8 9551309.7 10669914.8 ns

C ko04138 Autophagy - yeast 107013.4 244987.8 446302.2 89944.4 112700.3 216229.1 < 0.05

G ko04141 Protein processing in endoplasmic 1232664.2 2194663.8 2963658.1 1656735.1 1844876.2 3033791.7 ns

reticulum

C ko04142 Lysosome 2503257.3 3304929.7 5540461.6 1677325.0 3699799.7 4449026.8 ns

C ko04144 Endocytosis 0.0 64.9 434.3 0.0 44.1 182.0 null

C ko04146 Peroxisome 6466886.6 8092770.5 8813682.0 7199696.2 8230001.6 8842309.9 ns

E ko04151 PI3K-Akt signaling pathway 1112968.7 1271915.6 1604790. 21027434.0 1076148.4 1640053.1 ns

E ko04152 AMPK signaling pathway 1864212.2 2259935.2 2504734.0 2081906.4 2312381.6 2580391.3 ns

C ko04210 Apoptosis 991351.9 1490416.9 2627450.8 455939.5 1595226.9 2017349.6 ns

O ko04211 Longevity regulating pathway 1270925.0 1805910.6 2226621.9 1397655.4 2179763.1 2377425.9 ns

O ko04212 Longevity regulating pathway - worm 8064132.1 9920206.7 11745951.4 8287631.1 10439135.7 10814628.2 ns

O ko04213 Longevity regulating pathway – 3268264.0 3776619.6 4314205.8 3622334.3 4263817.4 4540253.8 ns

multiple species

C ko04214 Apoptosis – fly 1426468.3 1654796.7 1999261.6 1423973.3 1523991.2 1795285.2 ns

C ko04215 Apoptosis - multiple species 5335.6 22979.0 75829.0 5599.3 11033.0 23188.9 ns

C ko04216 Ferroptosis 3789544.4 4503950.7 5557814.7 3538969.3 4141152.8 4480121.1 ns

C ko04217 Necroptosis 5900382.8 7584389.7 8722946.9 5581013.6 7611088.1 8416857.4 ns

O ko04260 Cardiac muscle contraction 147161.2 305746.8 441444.8 869970.7 1183631.4 1452823.7 < 0.001

E ko04370 VEGF signaling pathway 0.0 0.0 0.0 0.0 0.0 0.0 null

E ko04371 Apelin signaling pathway 0.0 0.0 0.0 0.0 0.0 0.0 null

O ko04380 Osteoclast differentiation 0.0 0.0 0.0 0.0 0.0 0.0 null

C ko04530 Tight junction 0.0 0.0 0.0 0.0 0.0 0.0 null

O ko04610 Complement and coagulation cascades 0.0 0.0 0.0 0.0 0.0 0.0 null

O ko04612 Antigen processing and presentation 541066.9 1061608.3 1313315.6 882385.7 947762.5 1453932.5 ns

O ko04614 Renin-angiotensin system 373815.0 698756.7 1043681.0 503521.8 881661.3 1050340.5 ns

O ko04621 NOD-like receptor signaling pathway 3743257.0 4212886.1 4845598.1 3726273.6 4501620.7 4915523.8 ns

O ko04622 RIG-I-like receptor signaling pathway 53545.5 155424.2 273025.8 97828.4 136874.3 167180.6 ns

O ko04625 C-type lectin receptor signaling pathway 0.0 0.0 0.0 0.0 0.0 0.0 null

O ko04626 Plant-pathogen interaction 2152371.9 2548280.5 2789290.4 2637139.6 2839689.9 3186405.8 ns

O ko04657 IL-17 signaling pathway 541066.9 1061608.3 1313315.6 882385.7 947762.5 1453932.5 ns

O ko04659 Th17 cell differentiation 541066.9 1061608.3 1313315.6 882385.7 947762.5 1453932.5 ns

O ko04666 Fc gamma R-mediated phagocytosis 0.0 0.0 0.0 0.0 0.0 0.0 null

E ko04668 TNF signaling pathway 0.0 0.0 0.0 0.0 0.0 0.0 null

O ko04714 Thermogenesis 3379727.1 4151531.5 5043353.4 4142113.9 4765425.4 5410939.3 ns

O ko04721 Synaptic vesicle cycle 0.0 0.0 0.0 0.0 0.0 0.0 null

O ko04723 Retrograde endocannabinoid signaling 0.0 0.0 0.0 0.0 0.0 0.0 null

O ko04724 Glutamatergic synapse 3044213.9 3791647.5 4396517.1 2799744.2 3757101.9 4200713.5 ns

O ko04726 Serotonergic synapse 1445.6 3081.9 7954.0 1273.7 2952.5 10588.6 ns

O ko04727 GABAergic synapse 3067097.7 3806770.7 4412410.5 2801210.3 3780526.3 4204948.3 ns

O ko04728 Dopaminergic synapse 1445.6 3081.9 7954.0 1273.7 2830.3 10588.6 ns

O ko04910 Insulin signaling pathway 2415329.9 3189688.8 3698937.4 2194585.1 3426967.9 3745580.1 ns

O ko04912 GnRH signaling pathway 0.0 0.0 0.0 0.0 0.0 0.0 null

O ko04913 Ovarian steroidogenesis 0.0 0.0 0.0 0.0 0.0 0.0 null

O ko04914 Progesterone-mediated oocyte 541066.9 1061608.3 1313315.6 882385.7 947762.5 1453932.5 ns

maturation

O ko04915 Estrogen signaling pathway 541066.9 1061608.3 1313315.6 882385.7 947762.5 1453932.5 ns

O ko04916 Melanogenesis 0.0 85.1 572.5 0.0 0.0 0.0 null

O ko04917 Prolactin signaling pathway 669838.8 895432.0 1140630.4 695992.5 926126.8 1274235.4 ns

O ko04918 Thyroid hormone synthesis 2739547.9 3450272.0 4502081.1 2548902.1 3880431.8 4201658.4 ns

O ko04919 Thyroid hormone signaling pathway 1228.5 4213.0 25808.1 729.3 2081.1 3337.9 ns

O ko04920 Adipocytokine signaling pathway 3179470.8 3968479.1 4999499.6 2802951.5 3396192.5 4187450.0 ns

O ko04921 Oxytocin signaling pathway 0.0 0.0 0.0 0.0 0.0 0.0 null

O ko04922 Glucagon signaling pathway 7584289.5 9880444.7 12729580.8 6808114.2 9305465.9 10693633.5 ns

O ko04923 Regulation of lipolysis in adipocytes 0.0 0.0 0.0 0.0 0.0 0.0 null

O ko04924 Renin secretion 259.5 917.5 4038.8 500.0 1489.7 3905.5 ns

O ko04928 Parathyroid hormone synthesis, 0.0 0.0 782.5 0.0 0.0 858.0 null

secretion and action

H ko04930 Type II diabetes mellitus 2053124.9 2394906.2 2741938.3 1988506.7 2329566.0 2599921.3 ns

H ko04931 Insulin resistance 3793940.5 4736904.8 5418959.5 3148244.2 4697479.3 5328908.5 ns

H ko04932 Non-alcoholic fatty liver disease 199009.4 357915.1 492642.8 877118.1 1210734.7 1521503.3 < 0.001

(NAFLD)

H ko04934 Cushing syndrome 412890.1 518131.2 734176.3 530206.0 658943.1 839314.2 ns

H ko04940 Type I diabetes mellitus 2179467.9 2800786.1 3107016.6 2028793.8 2621677.7 2862901.9 ns

O ko04961 Endocrine and other factor-regulated 0.0 0.0 0.0 0.0 0.0 0.0 null

calcium reabsorption

O ko04962 Vasopressin-regulated water 0.0 0.0 0.0 0.0 0.0 0.0 null

reabsorption

O ko04964 Proximal tubule bicarbonate reclamation 618981.2 860445.2 1207413.7 478540.3 671992.3 842014.5 ns

O ko04966 Collecting duct acid secretion 0.0 0.0 0.0 0.0 0.0 0.0 null

O ko04972 Pancreatic secretion 0.0 0.0 0.0 0.0 0.0 0.0 null

O ko04973 Carbohydrate digestion and absorption 939464.9 1392582.7 1696235.9 679664.8 1351968.9 1512447.4 ns

O ko04974 Protein digestion and absorption 800880.3 1065661.1 1999118.0 498681.7 1126249.0 1390280.0 ns

O ko04976 Bile secretion 0.0 0.0 0.0 0.0 0.0 0.0 null

O ko04978 Mineral absorption 480697.7 730582.0 1159516.0 681587.9 1096601.4 1263044.1 ns

O ko04979 Cholesterol metabolism 1227.6 5306.1 19717.0 1208.2 3890.2 7255.1 ns

H ko05010 Alzheimer disease 2844565.0 3142382.9 3592889.5 3552460.2 3848214.5 4448248.9 < 0.01

H ko05012 Parkinson disease 199009.4 357915.1 492642.8 877118.1 1210734.7 1521503.3 < 0.001

H ko05014 Amyotrophic lateral sclerosis (ALS) 340097.7 469124.6 585874.3 714299.8 1037153.4 1291135.0 < 0.001

H ko05016 Huntington disease 1413993.7 1927484.2 2283180.4 2382919.2 2886831.1 3380090.2 < 0.01

H ko05020 Prion diseases 47525.8 97845.5 139550.8 119751.6 289716.2 391611.0 < 0.01

H ko05030 Cocaine addiction 1445.6 3081.9 7954.0 1273.7 2830.3 10588.6 ns

H ko05031 Amphetamine addiction 1445.6 3081.9 7954.0 1273.7 2830.3 10588.6 ns

H ko05034 Alcoholism 1445.6 3081.9 7954.0 1273.7 2830.3 10588.6 ns

H ko05100 Bacterial invasion of epithelial cells 82662.9 205086.0 409093.8 117279.2 222480.8 344954.5 ns

H ko05110 Vibrio cholerae infection 31994.6 68562.3 156168.8 136316.7 303333.7 587327.1 < 0.01

C ko05111 Biofilm formation - Vibrio cholerae 9654169.1 11362041.6 12112628.0 11018690.6 11896554.7 12877187.6 ns

H ko05120 Epithelial cell signaling in 2758197.3 3210844.0 4062597.4 3586786.3 4403595.4 6779689.5 < 0.05

Helicobacter pylori infection

H ko05131 Shigellosis 0.0 0.0 0.0 0.0 0.0 174.8 null

H ko05132 Salmonella infection 1129547.0 1538317.3 1876620.8 804628.4 1292622.5 1489905.6 ns

H ko05133 Pertussis 441397.1 933420.2 1134908.3 998436.4 1827591.8 3151010.0 < 0.01

H ko05134 Legionellosis 5566576.4 6702715.3 7697472.2 6167053.2 6810956.9 7480981.2 ns

H ko05140 Leishmaniasis 0.0 0.0 0.0 0.0 0.0 0.0 null

H ko05142 Chagas disease 122895.0 228171.0 413080.1 100010.5 158146.5 285517.8 ns

(American trypanosomiasis)

H ko05143 African trypanosomiasis 123654.7 228653.1 412080.2 87331.5 157842.0 285742.6 ns

H ko05145 Toxoplasmosis 5335.6 22979.0 75829.0 5599.3 11033.0 23188.9 ns

H ko05146 Amoebiasis 378154.0 562588.2 912845.2 277616.5 596663.2 843256.4 ns

H ko05150 Staphylococcus aureus infection 1155078.6 3162878.8 4326944.8 1584636.4 3140894.1 3660942.7 ns

H ko05152 Tuberculosis 5500647.4 7121375.3 7978496.6 4937554.1 6552562.6 7245639.9 ns

H ko05161 Hepatitis B 5335.6 22979.0 75829.0 5599.3 11033.0 23188.9 ns

H ko05163 Human cytomegalovirus infection 5335.6 23131.8 75829.0 5599.3 11033.0 23188.9 ns

H ko05164 Influenza A 13615.9 34103.6 94765.9 16754.6 25465.5 115766.4 ns

H ko05165 Human papillomavirus infection 2041346.8 2381343.7 2725148.7 1986169.2 2324028.0 2583939.8 ns

H ko05166 Human T-cell leukemia virus 1 infection 1227.6 5306.1 19717.0 1208.2 3890.2 7255.1 ns

H ko05167 Kaposi sarcoma-associated herpesvirus 5335.6 23131.8 75829.0 5599.3 11033.0 23188.9 ns

infection

H ko05168 Herpes simplex virus 1 infection 5335.6 22979.0 75829.0 5599.3 11033.0 23188.9 ns

H ko05170 Human immunodeficiency virus 1 5335.6 22979.0 75829.0 5599.3 11033.0 23188.9 ns

infection

H ko05200 Pathways in cancer 2055970.8 2413815.5 2808760.4 2372638.8 2676201.7 2930237.9 ns

H ko05202 Transcriptional misregulation in cancer 0.0 0.0 0.0 0.0 0.0 0.0 null

H ko05203 Viral carcinogenesis 2041346.8 2381343.7 2725148.7 1986169.2 2324028.0 2583939.8 ns

H ko05204 Chemical carcinogenesis 137040.3 242728.8 429651.5 201872.9 385708.2 569275.2 ns

H ko05205 Proteoglycans in cancer 2049000.9 2405410.0 2716990.8 2010730.3 2537050.2 2759063.1 ns

H ko05206 MicroRNAs in cancer 2782260.3 3482220.7 4168173.0 3568129.3 4184529.5 4400783.4 ns

H ko05210 Colorectal cancer 5335.6 22979.0 75829.0 5599.3 11033.0 23188.9 ns

H ko05211 Renal cell carcinoma 412890.1 518131.2 734176.3 530206.0 658943.1 839314.2 ns

H ko05215 Prostate cancer 541066.9 1061608.3 1313315.6 882385.7 947762.5 1453932.5 ns

H ko05219 Bladder cancer 30759.1 41686.7 57629.3 23817.1 38864.6 85138.4 ns

H ko05222 Small cell lung cancer 5335.6 23131.8 75829.0 5599.3 11033.0 23188.9 ns

H ko05225 Hepatocellular carcinoma 402150.1 734569.0 1062218.9 557726.8 795923.5 860114.5 ns

H ko05230 Central carbon metabolism in cancer 6734625.0 9038434.7 11304343.4 6799667.9 9015786.0 9963069.1 ns

H ko05231 Choline metabolism in cancer 148267.4 213195.0 331882.8 331885.1 608080.0 744998.9 < 0.001

H ko05322 Systemic lupus erythematosus 674.2 2588.8 7173.9 1655.7 3952.3 10635.4 ns

H ko05323 Rheumatoid arthritis 0.0 0.0 0.0 0.0 0.0 0.0 null

H ko05340 Primary immunodeficiency 2265949.4 2815010.2 3522929.9 1968812.7 3078443.1 3261878.0 ns

H ko05410 Hypertrophic cardiomyopathy (HCM) 259.5 917.5 4038.8 500.0 1489.7 3905.5 ns

H ko05416 Viral myocarditis 5335.6 22979.0 75829.0 5599.3 11033.0 23188.9 ns

H ko05418 Fluid shear stress and atherosclerosis 4767149.2 5708788.3 6558557.0 5444868.1 6140724.3 6449850.9 ns

M: Metabolism, G: Genetic Information Processing, E: Environmental Information Processing, C: Cellular Processes, O: Organismal Systems, H: Human Diseases. The Mann–Whitney *U* test was used to compare the abundance of KEGG pathways between the *H. pylori* negative and positive groups.

ns: nonspecific, null: the statistical significance test was invalid.
